# Enhancement of Supercapacitor Performance of Electrochemically Grown Nickel Oxide by Graphene Oxide

**DOI:** 10.3390/ma16083068

**Published:** 2023-04-13

**Authors:** Mohammad H. BinSabt, Ahmed Galal, Ahmed Abdel Nazeer

**Affiliations:** Chemistry Department, Faculty of Science, University of Kuwait, P.O. Box 5969, Safat 13060, Kuwait

**Keywords:** Ni-films/graphene oxide, supercapacitor, galvanostatic charge/discharge

## Abstract

β-Ni(OH)_2_ and β-Ni(OH)_2_/graphene oxide (GO) were prepared on an Ni foil electrode using the electrochemical cyclic voltammetry formed in 0.5 M KOH solution. Several surface analyses such as XPS, XRD, and Raman spectroscopies were used to confirm the chemical structure of the prepared materials. The morphologies were determined using SEM and AFM. The addition of the graphene oxide layer showed a remarkable increase in the specific capacitance of the hybrid. Through the measurements, the specific capacitance values were 280 F g^−1^ and 110 F g^−1^ after and before adding 4 layers of GO, respectively. The supercapacitor displays high stability until 500 cycles are charged and discharged almost without a loss in its capacitance values.

## 1. Introduction

Electrochemical supercapacitors benefited from the advantage of the advent of carbon-based materials and metal oxide hybrids. It is expected that an electrical double layer will be established at the interfacial sites of such hybrids which are associated with a fast electron transfer redox process [1,2]. On the other hand, metal oxides are related to a “pseudocapacitance” component and an intrinsic double layer capacitance from the carbon-based material [3,4]. Graphene has emerged as a leading candidate for use in electrochemical supercapacitors among carbon-based materials. Graphene electrodes in potassium hydroxide solutions were used to produce a specific capacitance of up to 135 F g^−1^ [5,6]. According to other investigations [7,8], graphene produces an extremely good long-life cycle of specific capacitance that retains up to 90% more than a thousand cycles, and has a specific capacitance of up to 117 F g^−1^.

Addressing the use of graphene in a 2D hybrid structure with metal oxides for such applications is of utmost importance. For instance, the specific capacitance of a decreased graphene oxide/SnO_2_ composite in sulfuric acid was increased noticeably to 34.6 F g^−1^. A graphene/ZnO composite film with a specific capacitance of 11.3 F g^−1^ was also presented as a supercapacitor contender in a different study [9,10]. SnO_2_/graphene was synthesized with the use of a microwave, and the resulting hybrid’s supercapacitor capabilities were evaluated [11,12]. For use in fast charge/discharge applications, a graphene/nickel oxide nanostructure composite was recently created and tested [13,14].

In this study, NiO in the mode of coralloid nanostructure has been deposited over graphene in 20% and 50% loading that displayed stable discharge capacities of 185 mAh/g and 450 mAh/g in both. Nickel oxide nanoparticles with graphene oxide were applied over glassy carbon electrodes and employed as a supercapacitor, displaying a 16-fold increase in capacitance [15,16].

Polycrystalline nickel surfaces go through three “stages” of phase transformations upon exposure to the appropriately applied potential in an alkaline medium. In this respect, Ni electrodes transit well-defined active to passive states, and eventually transpassive states when subjected to polarization potentials, such as *E*_p_, as follows: *E*_p_ ≤ 0.5 V; 0.5 V < *E*_p_ > 1.3 V; and *E*_p_ ≥ 1.3 V vs. normal hydrogen electrode (NHE). It was stated that the three transits correspond to the formation of the following phases: α-Ni(OH)_2_, β-Ni(OH)_2_, and NiOOH hydroxide/oxide, respectively [17,18]. The kinetics and mechanisms of the formation of α-Ni(OH)_2_ and NiOOH have already been extensively studied using different electrochemical methods, such as cyclic voltammetry, chronoamperometry, and electrochemical impedance spectroscopy, and the nature of surface films using ex-situ ultra-high vacuum (UHV) analyses [16,17,18,19,20,21] has also been studied.

It is widely known that passive layers formed on Ni in KOH causing the formation of Ni(OH)_2_ layers possess semiconductive properties. It was noticed that the capacitance of α-Ni(OH)_2_ deposits reduces with a thickness corresponding to insulating properties [16,17,20]. The study of the interaction of graphene with supporting or doping materials, which may provide an additional important degree of control of graphene properties, is an important growing research field [22,23,24,25]. Accordingly, the modification of the electronic structure of the graphene through interaction with substrates is of paramount importance. The development of electrochemically reversible systems for storing energy originated during the late years of the last century. These systems depend on the capacitance related with the charging and discharging of the double at the electrode interfaces. In this case, there are large interfacial capacities of many tens of farads per gram of active electrode materials such as “high-area” carbon powders, fibers, etc. These large specific-value capacitors are perceived as energy storage systems that can offer high power density in charge and discharge. Cycle lives to the order of 10^5^ to 10^6^ or higher can be achieved which make storage devices known as supercapacitors, developed and based on metal/carbon hybrids, much better compared to regular batteries.

In this work, β-Ni(OH)_2_ films are electrochemically deposited at a controlled thickness, and graphene oxide is deposited using chemical techniques, including the low-energy microwave irradiation over films of Ni-hydroxides/oxides. This is important in order to study the electrochemical/electronic properties of the resulting nano-hybrids, Ni-films/graphene oxide, as well as their applicability as supercapacitors. The Ni-substrate and its corresponding oxide/hydroxide/oxy-hydroxide are not suitably used in supercapacitor applications as a stand-alone surface. Several synthesis techniques have been used to modify the structure of NiO in order to improve its electrochemical performance in supercapacitor applications [13]. Therefore, in this study, we anticipate the modification of the nickel oxide layer with graphene oxide to improve the specific capacitance and performance of the hybrid as a candidate for supercapacitor application. The chemical structure of the prepared materials was confirmed by XPS, XRD, and Raman spectroscopy. To obtain these targets, a wide range of electrochemical and surface characterization techniques was used, e.g., cyclic voltammetry, cyclic charging/discharging, scanning electron microscopy (SEM), atomic force microscopy (AFM), and surface-enhanced Raman spectroscopy.

## 2. Materials and Methods

### 2.1. Materials

All chemicals and solvents (GO, KOH, ethanol, and acetone) were of analytical grade, procured from Sigma-Aldrich, and used as received without further purification.

### 2.2. Preparation of the Ni/β-Ni(OH)_2_ and Ni/β-Ni(OH)_2_/GO

The Ni/β-Ni(OH)_2_ layer was formed electrochemically using a cyclic voltammetry technique which employs an electrochemical potentiostat (Gamry Reference 3000) at room temperature. This was followed by the application of GO layers. The deposition was carried out from the three-electrode cell; the Ni foil represents the working electrode, while Ag/AgCl is the reference electrode in the presence of a Pt foil with the same dimensions of the working electrode, and is a counter electrode. The oxidation and the formation of hydroxide over the Ni foil were carried out from an electrolyte of 0.5 M KOH. EIS tests were carried out at room temperature (23 ± 1 °C) in the frequency range of 100 kHz to 0.2 Hz, with the AC signal measuring 5 mV peak-to-peak. To ensure reproducibility, all electrochemical experiments were repeated three times, and the average results with high reproducibility were considered.

GO was dispersed in KOH under microwave irradiation. The deposition of the GO layers on the Ni/β-Ni(OH)_2_ electrode was carried out by dropping 100 μL of 0.5 M GO solution on the surface of the electrode using microwave irradiation to dry the solvent and form a layer.

### 2.3. The Electrochemical Supercapacitor Measurements

The supercapacitor measurements were carried out through a three-electrode cell; the Ni/β-Ni(OH)_2_ or Ni/β-Ni(OH)_2_/GO represented the supercapacitor electrode, Ag/AgCl is the reference electrode, and a Pt foil with the same dimensions represented the counter electrode. The cyclic voltammetry and charge/discharge parameters are determined. The stability of the electrode and specific capacitance were determined by galvanostatic charge/discharge. The supercapacitor behavior is determined through an Ni(CH_3_COOH)_2_ electrolyte.

### 2.4. The Characterization

All the prepared materials are characterized using the scanning electron microscope, SEM (ZEISS LEO, Gemini Column, Carl Zeiss, Jena, Germany), and atomic force microscope, AFM XE-100E (PARK SYSTEM, Suwon, Republic of Korea). Moreover, the chemical structures are confirmed using X-ray photoelectron spectroscopy (XPS, VG Multilab 2000-X equipped). Using an AlKα source with spot sizes ranging from 200 to 850 μm, X-ray diffraction (XRD) was carried out at room temperature (at 2*θ* = 10–80°) using a Bruker AXS D8 advanced diffractometer which employs a copper target (λ = 1.5418 Å), DIFFRACplus software (https://www.bruker.com/en/products-and-solutions/diffractometers-and-x-ray-microscopes/x-ray-diffractometers/diffrac-suite-software.html (accessed on 12 February 2023)), and surface-enhanced Raman spectroscopy (SERS) XDR Raman.

## 3. Results and Discussion

### 3.1. Preparation of Ni/β-Ni(OH)_2_ and Ni//β-Ni(OH)_2_/GO Electrodes

**Figure 1:**
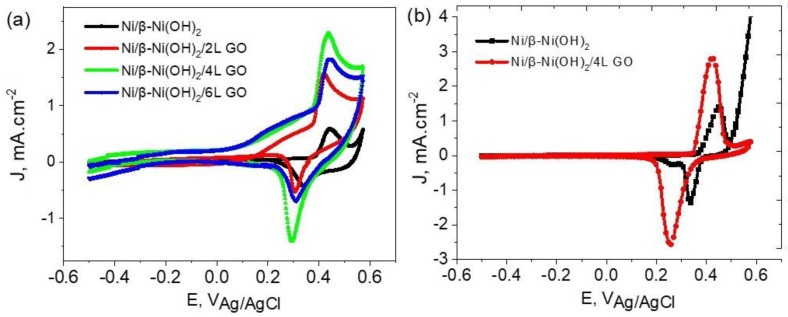
CVs for bare Ni/β-Ni(OH)_2_ and Ni/β-Ni(OH)_2_ coated with GO (2, 4, and 6 layers) for (**a**) 1st cycle and (**b**) 100th cycles in de-aerated 0.5 M KOH scan rate 50 mV s^−1^.

The cyclic voltammetry experiments were carried out at a scan rate 50 mV s^−1^ in the potential window of −0.5 V to +0.6 V (vs. Ag/AgCl). Figure 1 represents the cyclic voltammograms of poly-crystalline Ni electrodes in 0.5 M KOH. The anodic and cathodic peaks have been attributed to the irreversible formation of β-Ni(OH)_2_ and the reversible formation of β-Ni(OOH) [17]. The anodic and cathodic peak potentials are located at +0.47 V and +0.32 V, respectively. This redox couple is attributed to the reversible formation and reduction of β-Ni(OOH) [17]. It can be observed that the potential region preceding this redox peak couple is classified by a flat-like current plateau in the range between −0.50 V to +0.40 V for the “bare” nickel electrode. However, the Ni electrodes coated with graphene oxide display a more distinct behavior. A noticeable increase in current is observed as the potential exceeds the value of +0.10 V up to +0.40 V, while the redox couple of peaks are still maintained.

This current increase for Ni electrodes coated with graphene oxide could be attributed to a capacitive component associated with the charging of a double layer between the β-Ni(OOH) oxide and the graphene oxide layer. It is expected that it will be necessary to carry out experimental measurements that could help reveal the nature of the current in this region.

It was observed from the data in Figure 1a that the coverage of Ni with four layers of graphene oxide (Ni/4L GO) displayed the highest anodic and cathodic currents corresponding to the potential redox couples of β-Ni(OOH) oxide. Moreover, the current in the preceding potential region between +0.10 V and +0.40 V reached its maximum value. Therefore, the following measurements will be concerned with Ni electrodes coated with four layers of graphene.

Figure 1b displays a comparison between the CVs of bare Ni and Ni/β-Ni(OH)_2_/4 L GO for the first cycle and cycle number 100. In each Figure, the current attributed to the GO-coated electrode is noticeably higher compared to the bare Ni electrode for the formation of Ni/β-Ni(OH)_2_ and Ni/β-Ni(OH)_2_/GO. From the results it is concluded that the Ni/β-Ni(OH)_2_ layer allows partial charge storage. The electrochemical formation of the/β-Ni(OH)_2_ layer is essential to improve its capacitance due to its thin structure and the appearance of the nano-dimensions of the oxide. Carbon-based materials are known for their relatively high conductivity and stable structure that allows for high charge storage ability [26]. The further application of multi-layers of GO in the form of crumpled sheets proved to be efficient as an active material that increases the charge storage ability of the hybrid.

### 3.2. Surface Morphology

**Figure 2:**
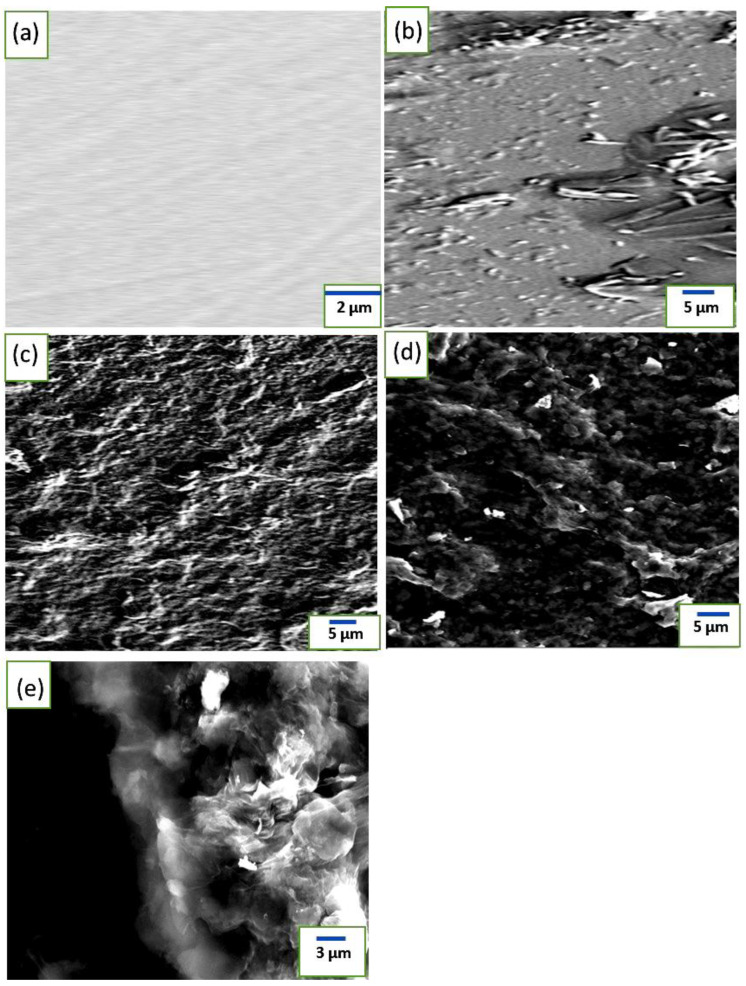
SEM micrographs for (**a**) polished Ni bare, (**b**) Ni/β-Ni(OH)_2_, (**c**) Ni/β-Ni(OH)_2_/4L GO before exposure, (**d**) Ni/β-Ni(OH)_2_/4L GO after exposure, and (**e**) cross-section of Ni/β-Ni(OH)_2_/4L GO.

**Figure 3:**
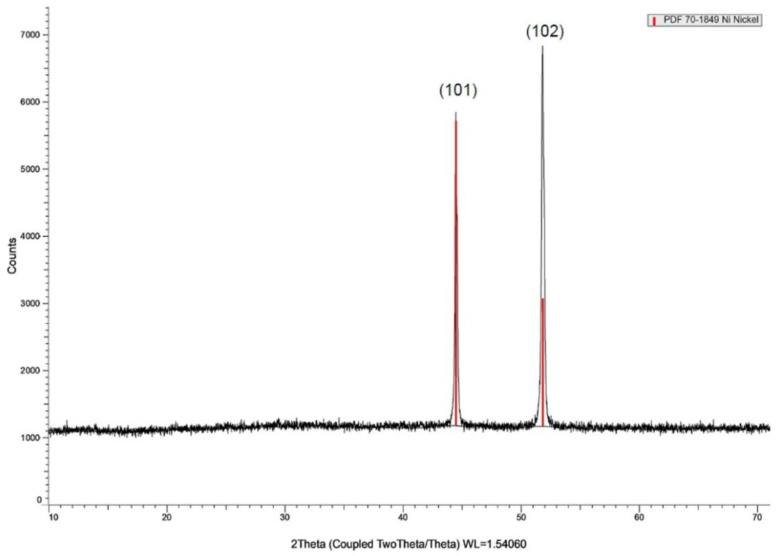
X-ray diffraction (XRD) pattern of Ni/β-Ni(OH)_2_.

Figure 2 shows the scanning electron micrographs of bare Ni, Ni/β-Ni(OH)_2_, and Ni/β-Ni(OH)_2_ coated with four layers of GO surfaces before and after exposure to 0.5 M KOH. Figure 3 shows the XRD pattern of the β-Ni(OH)_2_ prepared electrochemically as previously explained. The XRD pattern shows sharp and symmetrical peaks of (101), and (102) planes around 2*θ* values of 44 and 52, respectively. This result is in good agreement with values previously reported. [27] As depicted in Figure 2b, after a polarization experiment between the potential limits of −0.5 V to +0.6 V, the surface of the electrode showed some roughness due to the formation of the corresponding Ni/β-Ni(OH)_2_. In Figure 2c, the Ni/β-Ni(OH)_2_/4L GO layer shows a compact film structure on the Ni surface. When the surface is subjected to the polarization step, a denser structure appears as depicted in Figure 2d due to the formation of the nickel hydroxide layer. The porosity of the oxide and its morphological structure should also be considered, since the interfacial structure between the nickel oxide phase and the graphene oxide is of paramount importance to affect the capacitive nature of the hybrid. When the layer of GO is applied to the formed nickel oxide phase, the pores allow the existence of various defects that should result in an increase in charge accumulation at the interface. Figure 2e shows a field emission electron microscope image of the interface between a β-nickel oxide and a four-layer formed graphene oxide.

**Figure 4:**
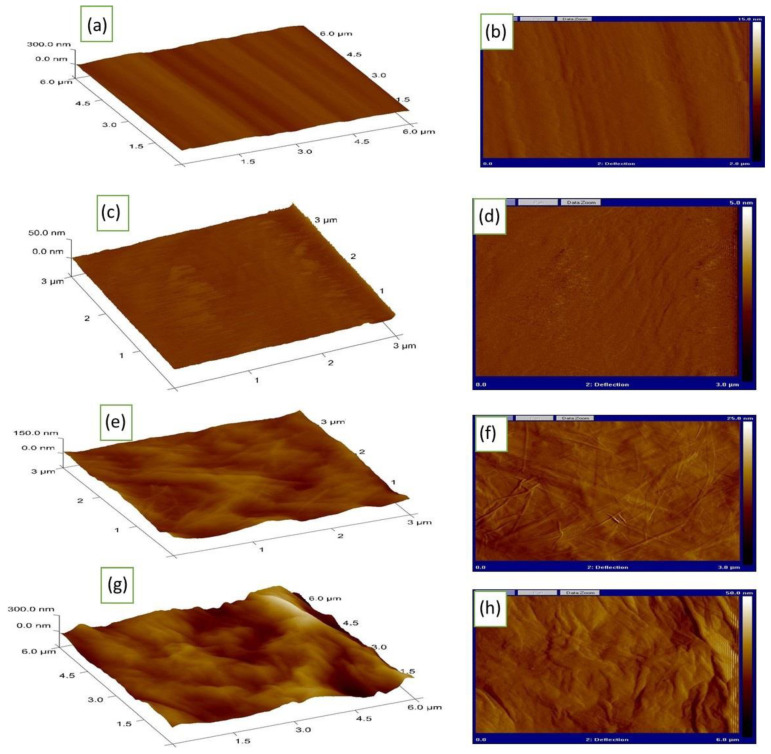
AFM graphs for (**a**,**b**) polished Ni bare, (**c**,**d**) β-Ni(OH)_2_, (**e**,**f**) Ni/β-Ni(OH)_2_/4L GO before exposure, and (**g**,**h**) Ni/β-Ni(OH)_2_/4L GO after exposure.

Atomic force microscopy (AFM) was also conducted to evaluate the surface roughness upon exposure to a potassium hydroxide solution and coverage with the graphene oxide layer. The texture features of the surface reflect the graduation in its roughness. Thus, the polished Ni electrode in Figure 4a,b showed the least roughness compared to the same after exposure to 0.5 M KOH with polarization forming Ni/β-Ni(OH)_2_ in Figure 4c,d. The formation of film layers over the surface of Ni indicates the formation of an oxide film with an increase in the relative surface roughness. The AFM micrograph of a graphene oxide-covered nickel oxide surface showed an elevation in the surface texture that indicates the full coverage of the surface of the substrate in Figure 4e,f. Upon exposure to 0.5 M KOH, the graphene oxide layer showed an increase in the elevation of the surface roughness in Figure 4g,h (Ni/β-Ni(OH)_2_/4L GO). These AFM figures confirm the behavior of materials as shown previously by SEM. Table 1 illustrates the surface roughness estimated from AFM. The polished Ni surface has an average surface roughness (Ra) of 2.39 ± 0.05 nm, while the Ra of β-Ni(OH)_2_ is 7.72 ± 0.08 nm. Furthermore, the roughness in the case of Ni/β-Ni(OH)_2_/4L GO before exposure to 0.5 M KOH equaled 22 ± 0.23 which increased to (Ra = 74 ± 0.31 nm) after exposure to 0.5 M KOH.

**Figure 5:**
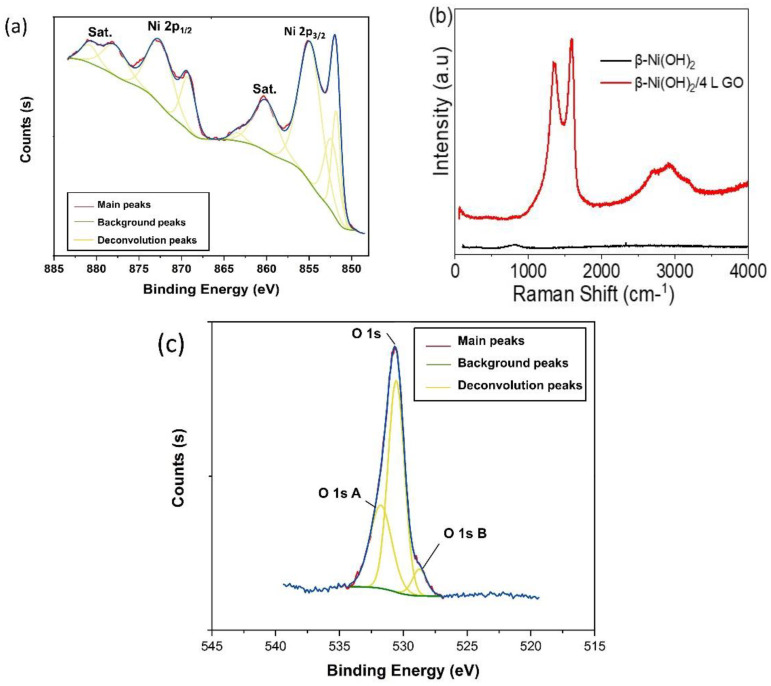
(**a**) XPS spectra of β-Ni(OH)_2_, (**b**) the Raman spectra for β-Ni(OH)_2_ and β-Ni(OH)_2_/4L GO, and (**c**) XPS spectra of O 1s.

The surface structure was examined by X-ray photoelectron spectroscopy (XPS) and surface-enhanced Raman spectroscopy (SERS). Figure 5a depicts the XPS spectra of Ni, showing several peaks at chemical shifts corresponding to the binding energies of Ni hydroxide species. Thus, the spectra recorded show the main Ni 2p_3/2_ peak at ca. 855 eV, which is attributed to Ni^2+^ [25]. At higher binding energies (ca. 860 and 881 eV), satellite peaks are observed; such peaks were previously cited [25,28]. Thus, when a thin hydroxide layer is formed on the electrode surface, we observe a small shoulder at the lower BE of the main Ni(II) peak in the Ni spectrum (ca. 852 eV) that corresponds to the metallic Ni electrode [25]. The peak of the Ni(II) appears at 862 eV. The peak at 873 eV can be assigned to Ni hydroxide. Moreover, the O 1s XPS spectra in Figure 5c display a peak at about 532 eV, suggesting that the oxygen is linked to nickel in the form of Ni(OH)_2_.

On the other hand, the successful formation of the graphene oxide film was confirmed from the Raman spectra given in Figure 5b. The 2 peaks appearing at 1340 and 1549 cm^−1^ correspond to the D and G bands, respectively, with the expected ratio.

### 3.3. Supercapacitor’s Nature of Nickel Oxide/Graphene Oxide Hybrid

**Figure 6:**
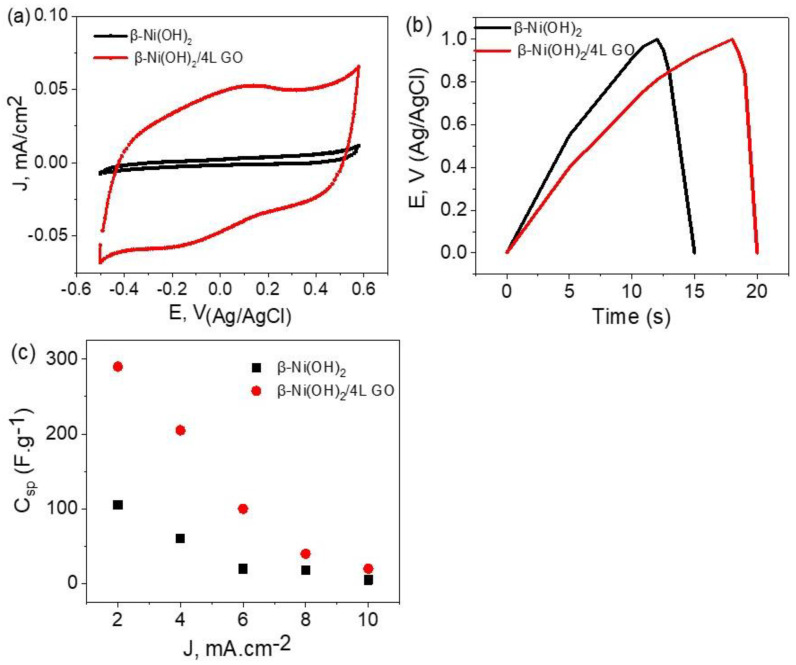
(**a**) cyclic voltammetry, (**b**) charge/discharge, and (**c**) specific capacitance for Ni/β-Ni(OH)_2_ and Ni/β-Ni(OH)_2_/4L GO electrode supercapacitor 0.01 M nickel acetate (cycle 100) at scan rate 50 mV s^−1^.

The capacitive nature of the nickel oxide/graphene oxide hybrid was further investigated in the 0.01 M nickel acetate electrolyte in Figure 6. The cyclic voltammetry (CV) diagrams were performed in the potential range of −0.5 V to +0.6 V vs. Ag/AgCl with a scan rate of 50 mV/s. The shape of the CV loops is close to a rectangular form that reflects the capacitive nature of the hybrid electrode [29]. The surface area of the electrode is about 0.785 cm^2^. Moreover, there is a great enhancement in the current values after coating the β-Ni(OH)_2_ with four layers of GO. The charge/discharge behavior of β-nickel oxide covered with 4 layers of graphene oxide was studied using chronopotentiometry from −0.0 V to 1.1 V at a constant current of 2 mA as depicted in Figure 6b. In the same manner, there are enhancements in the charge and discharge behavior after coating the β-Ni(OH)_2_ with four layers of GO; this indicates the enhancements in the charge accumulation on the electrode due to the charges on the GO material. It is important to notice that there is no indication of the internal resistance of the electrode that may cause an IR drop. This could be attributed to the well-established interfacial connection between the nickel oxide/graphene oxide on the electrode side and the electrode surface/electrolyte. A previous study showed that graphene oxide possesses remarkable energy storage capacity [30]. In this study, graphene oxide was proved to improve the capacitance to reach 423 F g^−1^ compared to graphite with relatively low capacitance. This proves that “standing alone GO” has a charge storing capacity that should be additive, and improve the performance of the underlying Ni-based oxide.

It is clear that in Figure 6c, the capacitive loop for the β-Ni(OH)_2_/4L GO is noticeably larger than that of the nickel oxide. The specific capacitances of β-nickel oxide and graphene-coated β-nickel oxide are 280 F g^−1^ and 110 F g^−1^, respectively. At this stage, it could be concluded that the specific capacitance contribution from four layers of graphene oxide is about two times that from β-nickel oxide as calculated from the galvanostatic cyclic charging/discharging measurements.

The specific capacitance, *C_sp_*, was calculated from the equation Equation (1) [31]; *I* is the charging current, ν is the scan rate, and m is the mass of coating.

(1) $$C_{sp}=\frac{2I}{\upsilon \times m}$$

**Figure 7:**
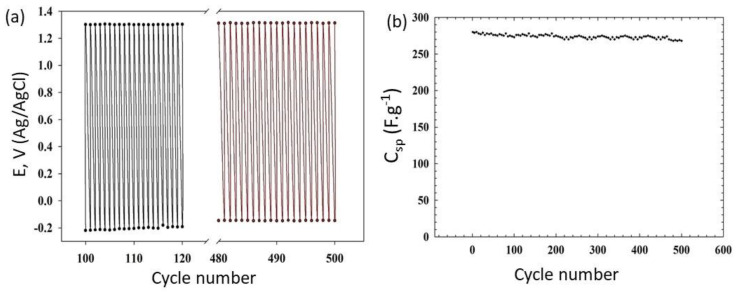
(**a**) Repeated charge/discharge cycles (50 cycles) and (**b**) The stability of the specific capacitance of Ni/β-Ni(OH)_2_/4L GO.

Since stability and reversibility are also crucial to the application of such materials in the supercapacitor industry [32], the repeated charge/discharge behavior of the two surfaces was also examined. Figure 7a shows the cyclic performance of the β-nickel oxide covered with 4 layers of graphene oxide as examined by galvanostatic charge/discharge for 500 cycles. It was noticed that the capacitance of β-nickel oxide covered with four layers of graphene oxide calculated from the data in Figure 7a exhibited almost no decay during and after the test completion, which indicates that the hybrid displays excellent long-term charge/discharge recycling ability in Figure 7b. Table 2 displays a comparison of the specific capacitance of different Ni-based oxides and their percentage of retention. The comparison shows that the specific capacitance for the thin film of β-Ni(OH)_2_ covered with four layers of GO is not as high compared with other materials given in the literature. However, all cited work did not mention the type or amount of NiO used. In addition, the retention percentage of the charge is higher for the present hybrid film compared to other materials.

## 4. Conclusions

This study presents very interesting results for supercapacitor preparation and measurements depending on Ni/β-Ni(OH)_2_ and Ni/β-Ni(OH)_2_/4L GO. The preparation of β-Ni(OH)_2_ over the Ni electrode was carried out through the cyclic voltammetry deposition from 0.5 M KOH. The XPS, XRD, and Raman confirmed the chemical structure of the prepared materials. From the supercapacitor measurements, the specific capacitance increased to 280 F g^−1^ after coating the electrode with 4 layers of GO. The supercapacitor displays stability and reproducibility until 500 cycles without any loss in capacitance. Our research may provide a reference and basis for the design and comparison of electrode materials for high-performance supercapacitors. Further optimization of phases and improvement of electrochemical performance are needed for future studies, as well as comparing the result based on supercapacitor application.

## Figures and Tables

**Table 1:** Surface roughness of the examined surfaces determined from AFM.

Sample	Average Surface Roughness
Polished Ni surface	2.39 ± 0.05
β-Ni(OH)_2_	7.72 ± 0.08
Ni/β-Ni(OH)_2_/4L GO before exposure	22 ± 0.23
Ni/β-Ni(OH)_2_/4L GO after exposure	74 ± 0.31

**Table 2:** Comparison of the specific capacitance of different Ni-based oxides and their percentage of retention.

Electrode Material	Specific Capacitance (F g^–1^)	Current Density (A cm^−2^)	Retention (%)	Reference
NiO/Carbon	849	3	78	[33]
NiO/Poly(Aniline)	936	1	86	[34]
NiO/MWCNT	1200	5	-	[35]
Co_3_O_4_/NiO/Graphene Foam	766	1	86	[36]
NiO/N-doped Carbon	850	1	-	[37]
Ni/β-Ni(OH)_2_/GO	280	2	97	This work

## Data Availability

The data are available on reasonable request from the corresponding author.
